# It Is Not Just in Faces! Processing of Emotion and Intention from Biological Motion in Psychiatric Disorders

**DOI:** 10.3389/fnhum.2018.00048

**Published:** 2018-02-08

**Authors:** Łukasz Okruszek

**Affiliations:** Institute of Psychology, Polish Academy of Sciences, Warsaw, Poland

**Keywords:** biological motion, schizophrenia, affective disorders, eating disorders, anxiety disorders, neurodegenerative diseases, social neuroscience, emotion recognition

## Abstract

Social neuroscience offers a wide range of techniques that may be applied to study the social cognitive deficits that may underlie reduced social functioning—a common feature across many psychiatric disorders. At the same time, a significant proportion of research in this area has been conducted using paradigms that utilize static displays of faces or eyes. The use of point-light displays (PLDs) offers a viable alternative for studying recognition of emotion or intention inference while minimizing the amount of information presented to participants. This mini-review aims to summarize studies that have used PLD to study emotion and intention processing in schizophrenia (SCZ), affective disorders, anxiety and personality disorders, eating disorders and neurodegenerative disorders. Two main conclusions can be drawn from the reviewed studies: first, the social cognitive problems found in most of the psychiatric samples using PLD were of smaller magnitude than those found in studies presenting social information using faces or voices. Second, even though the information presented in PLDs is extremely limited, presentation of these types of stimuli is sufficient to elicit the disorder-specific, social cognitive biases (e.g., mood-congruent bias in depression, increased threat perception in anxious individuals, aberrant body size perception in eating disorders) documented using other methodologies. Taken together, these findings suggest that point-light stimuli may be a useful method of studying social information processing in psychiatry. At the same time, some limitations of using this methodology are also outlined.

## Introduction

It has recently been highlighted that the field of social neuroscience offers a number of techniques that can be effectively used for studying the processes that may underlie reduced functioning of psychiatric patients (Cacioppo et al., 2014; Fett et al., 2015; Ibáñez et al., 2016). Social cognitive deficits are found in various psychiatric populations (Samamé et al., 2012; Savla et al., 2013; Plana et al., 2014; Weightman et al., 2014) and may be of great importance for patients’ functional capacity (Fett et al., 2011). Although a wide range of techniques can be used to examine emotion recognition and theory of mind in patients, a substantial proportion of studies have examined the processing of social information conveyed by static displays of human faces or eyes (Savla et al., 2013). While the use of these types of stimuli is well-established in social cognitive studies, the static nature of the stimuli limits the ecological validity of this measurement method, given the dynamic nature of social cognitive processes. To overcome this problem, one may utilize videoed vignettes of actions and/or interactions of real-life social agents for social cognitive examination (McDonald et al., 2003; Dziobek et al., 2006). However, for such complex stimuli to be correctly processed a wide range of both verbal and non-verbal signals (facial and bodily movements, gaze direction, prosody, proximity between the agents) must be taken into consideration. Thus, patients’ inability to process these types of stimuli correctly reflects a wide variety of underlying social cognitive problems. Furthermore, perception of either static or dynamic full displays of real-life actors may be affected by numerous confounding factors, e.g., likeability of the agent presented or cultural factors (Mehta et al., 2011).

Minimalistic, dynamic, point-light displays (PLDs) may be a viable alternative for presenting social information while avoiding the problems that can afflict studies that use static or dynamic full displays of agents. Since the introduction of point-light motion methodology to the field of experimental psychology, by Swedish psychologist Gunnar Johansson (Johansson, 1973), numerous researchers have used it to show that the human visual system is finely tuned to decipher information on the gender, physical characteristics, affective state, or intention of the person presented (see Troje, 2013) for a review of studies on biological motion perception in healthy individuals). Furthermore, the presentation of whole-body motion that is visually downgraded to several point-lights attached to the main joints and limbs of the body, may be a culturally unbiased way to study social information processing (Pica et al., 2011).

In addition, the pattern of neural activity and connectivity during the processing of PLDs may be, to some extent, similar to that observed when processing other forms of social agent presentation (faces, animated shapes; Dasgupta et al., 2017). Processing of the whole-body motion from PLDs is strongly linked to the posterior superior temporal sulcus (pSTS) activation, which is mostly lateralized to the right hemisphere (Van Overwalle and Baetens, 2009). At the same time, face-processing network includes occipital and fusiform face areas, posterior and anterior STS, as well as amygdala and insula (Duchaine and Yovel, 2015). Furthermore, while processing of both types of the stimuli strongly engage pSTS, (Deen et al., 2015) observed that, despite significant overlap, pSTS responses to faces and PLDs may differ reliably, with face-sensitive pSTS region being placed slightly anterior to region responding to biological motion.

While a large body of research was devoted to the study of various aspects of face perception across psychiatric disorders, knowledge of emotion or intention processing on the basis of biological motion processing in patients is relatively scarce. This may be a little surprising, especially given the amount of attention that biological motion processing received in the field of neurodevelopmental disorders (Pavlova, 2012, 2017). Thus, this article aims to provide a review of findings on recognition of emotion or intention from biological motion across psychiatric disorders.

## Methods

A PubMed search using terms “(“biological motion” or “point-light motion”) and (“emotion” or “intention”)” was performed to identify studies for the current mini-review. Additionally the search was supplemented by relevant articles found by reviewing the references provided in the identified articles. Relevant studies have been grouped accordingly to major categories from ICD-10 “Mental Behavioral and Neurodevelopmental disorders” section. Given that the studies on biological motion processing in autism spectrum disorders and other developmental disorders were reviewed in Pavlova (2012), and more recently in Pavlova (2017), findings from this areas are not discussed in the current review. Additionally, description of the commonly used PLD tasks has been provided in the “*Tasks*” section.

## Tasks

Most of the studies which examined processing of emotion from PLDs in psychiatric populations (Schizophrenia: Bigelow et al., 2006; Couture et al., 2010; Henry et al., 2010; Brittain et al., 2012; Kern et al., 2013; Vaskinn et al., 2016; Bipolar Disorder: Vaskinn et al., 2017; MDD: Loi et al., 2013; Eating Disorders: Zucker et al., 2013; Lang et al., 2015; Dapelo et al., 2017; Alzheimer’s Dementia: Henry et al., 2012) utilized stimuli developed by Heberlein et al. (2004). During the Emotion from the Biological Motion task (EBM) participant observes a single point-light agent walking across the screen and his/her task is to select the alternative which best describes agent’s affective state (happiness, sadness, fear, anger, neutral). Another set of stimuli for investigating emotion recognition in dyadic and monadic PLDs was developed by Lorey et al. (2012) and effectively applied to investigate social cognitive processes in psychiatric populations (Kaletsch et al., 2014a,b).

Two tasks were used to investigate intentions inference from PLDs across psychiatric populations. During the Gesture Perception Task (GPT; Jaywant et al., 2016b) participant is presented with single PLD (Zaini et al., 2013) and has to: (i) classify gesture performed by PLD as either communicative or non-communicative; and (ii) verbally describe each action. Alternatively, Communicative Interaction Database Five Alternative Forced Choice task (CID-5; Manera et al., 2015) present 21 dyadic PLDs and requires participant to: (i) decide if agents communicated or acted independently; and (ii) to identify the correct action description among the five alternatives.

## Neurodegenerative Disorders

Two studies examined the ability to recognize emotion from PLDs in patients with Alzheimer’s dementia (Henry et al., 2012; Insch et al., 2015). The first (Henry et al., 2012) observed that while deficits in facial emotion recognition can be found both in patients with AD and in individuals with mild cognitive impairment (MCI), deficient EBM performance was observed only in patients with AD. This observation was further corroborated by Insch et al. (2015), who found decreased performance in emotion recognition from PLDs in older adults, which was further reduced in patients with AD.

Another line of studies (Jaywant et al., 2016a,b) examined biological motion processing in individuals with Parkinson’s disease (PD). Interestingly, patients with PD demonstrated reduced sensitivity to biological motion (Jaywant et al., 2016a) and recognition of non-communicative, object-oriented gestures (Jaywant et al., 2016b), but did not differ from healthy controls when describing communicative gestures in GPT (Jaywant et al., 2016b).

## Schizophrenia

Patients with schizophrenia (SCZ) present deficits across multiple domains of the biological motion processing, including biological vs. scrambled motion discrimination (Kim et al., 2005, 2011, 2013; Kern et al., 2013; Jahshan et al., 2015) and detection of masked biological motion (Hastings et al., 2013; Spencer et al., 2013; Matsumoto et al., 2015, 2017). For a detailed discussion of the behavioral and neural correlates of biological motion processing in SCZ, please refer to our recent systematic review and meta-analysis of studies in this area (Okruszek and Pilecka, 2017). A sub-meta-analysis of six studies that assessed EBM performance (Bigelow et al., 2006; Couture et al., 2010; Henry et al., 2010; Brittain et al., 2012; Kern et al., 2013; Vaskinn et al., 2016) revealed moderate to large (*d* = 0.61) deficits in SCZ. Thus, while still impaired, this domain of social cognition differentiates SCZ from healthy controls to a lesser extent than does facial emotion identification (*d* = 0.89; Kohler et al., 2010) or emotional prosody processing (*d* = 1.24; Hoekert et al., 2007). Furthermore, links have been found between recognition of emotion from biological motion and higher-order social perception (Brittain et al., 2012), facial emotion identification and empathic accuracy (Olbert et al., 2013), and neurocognition and functional capacity (Engelstad et al., 2017).

Furthermore, we have shown that SCZ display reduced ability to explicitly categorize actions of dyadic PLDs as either communicative or individual in CID-5 (Okruszek et al., 2015). However, we have recently observed that despite biological motion processing deficits, SCZ are still able to use information carried by a communicative action of one agent to predict the action of the other agent (“interpersonal predictive coding”; Okruszek et al., 2018). Furthermore, similar perceptual biases were elicited in SCZ and in healthy controls by observing communicative gestures of one agent during PLD-based simultaneous masking detection task (Okruszek et al., 2017a). These findings, suggesting intact interpersonal predictive coding in SCZ were congruent with our recent functional neuroimaging results (Okruszek et al., 2017b): reduced activity and functional connectivity of the right pSTS, but similar action observation network activity were observed in SCZ compared with healthy controls during processing of communicative interactions vs. individual actions of dyadic PLDs (Okruszek et al., 2017b).

## Affective Disorders

While recognition of biological motion appears intact in patients with major depressive disorder (Kaletsch et al., 2014b), studies of emotion recognition from PLDs have revealed the same mood-congruent biases in patients when processing biological motion using other types of social stimuli, i.e., faces (Bourke et al., 2010) or verbal prosody (Péron et al., 2011; Loi et al., 2013). Using EBM, Loi et al. (2013) found that patients with depression exhibit a deficit in the recognition of happiness, but not of anger, sadness, fear, or neutral states, compared with both patients with depression in remission and healthy controls with no history of depression. On the other hand, Kaletsch et al. (2014b) observed that patients with MDD rate negative (but not positive) dyadic interactions presented in PLDs as more negative and more intense than do healthy controls.

Recently, a small but significant (*d* = 0.40) impairment in EBM was documented in patients with bipolar disorder (Vaskinn et al., 2017). No differences were observed between patients with type I and type II BD, or between patients with and without a history of psychosis. Furthermore, unlike the MDD group, patients with BD showed a similar extent of impairment for all emotions and no mood-congruent biases, and no association between impairments and either depressive or manic symptomatology.

## Anxiety Disorders

It has been documented that depth-ambiguous displays of biological motion are more often interpreted as being oriented toward rather than away from the viewer, even when both interpretations are equally plausible (Vanrie et al., 2004). This effect was termed “facing-the-viewer bias” and is usually explained by the preposterous consequences associated with mistaking an approaching agent for a retreating one, and thus may be interpreted as the impact of top-down factors (e.g., attribution of hostile intentions) on perception. One of the factors that has been shown to affect susceptibility to facing-the-viewer bias during the perception of a bistable point-light walker is the level of anxiety in an individual (Van de Cruys et al., 2013; Heenan and Troje, 2014; Heenan et al., 2014). Furthermore, facing-the-viewer bias has been found to be reduced by physical exercise and an anxiety-reducing task (progressive muscle relaxation; Heenan and Troje, 2014). Interestingly, the opposite bias (interpreting the walker as facing away from the observer) was observed in individuals with high levels of social anxiety, which can be interpreted in terms of “wishful seeing” and protecting oneself (Van de Cruys et al., 2013). Facing-the-viewer bias has also been found to be mediated by inhibitory abilities in individuals with high social anxiety (Heenan and Troje, 2015).

## Eating Disorders

The main focus of studies using biological motion stimuli to study social perception in eating disorders has been abilities associated with processing the weight or BMI of the agent. Individuals with either anorexia nervosa (AN; Phillipou et al., 2016) or bulimia nervosa (BN; Vocks et al., 2007) were shown to display abnormal processing of the body size of PLDs. When it comes to emotion processing, two studies examined EBM performance in individuals with AN (Zucker et al., 2013; Lang et al., 2015). Zucker et al. (2013) found overall worse recognition of emotion from biological motion by patients with AN compared with both healthy controls and weight-restored (≥12 months) individuals with AN. Deficient EBM performance was associated with symptom severity as measured by self-reported dietary restraint in patients. Moreover, analyses of the recognition of specific emotions revealed that individuals with AN attributed more anger and less sadness to the PLDs than controls and weight-restored individuals with AN. No differences were found, however, for the remaining categories (fear, happiness, neutral). These results were partially replicated by Lang et al. (2015), who found decreased recognition of sadness from PLDs in a well-powered (*n* = 97) sample of females with AN compared with healthy controls. Furthermore, overall worse recognition of emotion conveyed by biological motion was observed in adolescent individuals with AN compared with demographically matched controls (Lang et al., 2015). Finally, emotion recognition from faces and point-light motion was recently compared in individuals with AN and BN by Dapelo et al. (2017), who found specific impairment in processing emotion from faces in both groups of individuals with eating disorders, but no differences in EBM performance between patients and healthy controls.

## Personality Disorders

Reduced recognition of emotion from whole-body motion was recently documented in healthy participants with elevated levels of traits associated with positive schizotypy syndrome (Blain et al., 2017). At the same time, no differences were found between patients with borderline personality disorder (BPD) and healthy controls in recognition of affective states from PLDs (Kaletsch et al., 2014a).

## Conclusion

This review focused on the application of biological motion methodology to the study of emotion or intention inference in patients with psychiatric disorders. Two main conclusions may be drawn from the current review. First, the social cognitive problems found in most of the psychiatric samples using PLDs were of smaller magnitude than that found for other methods of social stimuli presentation (e.g., face, voice; SCZ: Okruszek and Pilecka, 2017; BD: Vaskinn et al., 2017; AN/BN: Dapelo et al., 2017; BPD: Kaletsch et al., 2014a). It has been suggested that the contribution of body motion to processing information about a person may be particularly important when viewing conditions are suboptimal or the person is viewed at a distance (Yovel and O’Toole, 2016). Correct identification of a person’s affective state or intention prior to a close proximity encounter may be crucial for one’s survival, thus the extraction of such information from biological motion may be one of our most basic and evolutionarily oldest social cognitive abilities. Furthermore, given the extensive neural networks that mediate processing of the human face (Haxby et al., 2000), recognition of emotion or intention from biological motion may be less affected by abnormal brain functioning in patients, compared with the processing of social information coming from other modalities. Direct support for this suggestion comes from the neuropsychological observations of body-face dissociation in emotion recognition in patients with limbic lesions, who were shown to be able to correctly recognize whole-body expressions, even despite alterations in facial affect processing (Sprengelmeyer et al., 2010; Atkinson et al., 2012). Additionally, while decreased facial emotion recognition was observed in both MCI and AD, decreased emotion processing from PLDs is observed only in patients with fully developed AD (Henry et al., 2012). Additionally, even though numerous studies documented decreased intention attribution in psychiatric patients (Fett et al., 2015), intact recognition of communicative interactions from both single (Jaywant et al., 2016b) and dyadic (Okruszek et al., 2015) PLDs was found in patients. Furthermore, intact interpersonal predictive coding was observed in SCZ with paradigms presenting dyadic PLDs (Okruszek et al., 2017a, 2018). Thus, studies that aim to examine the mechanisms associated with the processing of social information in psychiatric disorders may benefit from combining standard methodologies (e.g., recognition of emotion or intention from static displays of faces) and dynamic PLD-based tasks.

The second main conclusion of the current review is the fact that specific social cognitive biases that have previously been observed using other methods (e.g., mood-congruent bias in MDD, Loi et al., 2013; Kaletsch et al., 2014b), increased threat perception in individuals with elevated anxiety (Heenan and Troje, 2015), aberrant body size perception in eating disorders (Vocks et al., 2007; Phillipou et al., 2016) can also be found in studies using PLDs. Thus, even though the information presented in PLDs is extremely limited, the stimuli are sufficient to elicit disorder-specific, social cognitive biases. Recognition of basic emotions conveyed by biological motion has been found to be relatively unaffected by cultural factors (Parkinson et al., 2017), thus PLDs may be effectively employed to study cross-cultural factors affecting social functioning in psychiatric populations (Mohan et al., 2016).

Taken together, these observations suggest that PLDs may be used as an additional source of information on social cognitive processes, especially when combined with other forms of social information presentation. One way to accomplish this may be by using multimodal stimuli that combine PLDs with auditory stimuli (Piwek et al., 2015). Furthermore, a wide variety of PLD tasks is readily available, some of which have already been shown to have satisfactory psychometric values (Kern et al., 2013). Finally, Shi et al. (2017) recently presented a Kinect-based method that allows one to produce PLDs without having access to a full motion-capture laboratory. In this way, point-light stimuli can be tailored to the specific needs of a study using low-cost and user-friendly methods.

While the benefits of using PLDs have been listed above, some drawbacks of this approach should also be mentioned. First, PLD-based tasks may have limited test-retest reliability, thus may not be suitable for longitudinal assessments (Kern et al., 2013). Second, none of the abovementioned tasks has undergone a standardization procedure, which limits their usefulness for clinical practice. Finally, knowledge of the neural markers of biological motion processing abnormalities in psychiatric populations is severely limited, especially when compared with the extensive literature on facial affect processing in psychiatric disorders.

## Author Contributions

ŁO reviewed the literature and wrote the manuscript.

## Conflict of Interest Statement

The author declares that the research was conducted in the absence of any commercial or financial relationships that could be construed as a potential conflict of interest.
